# Meat Composition and Quality Assessment of King Scallops (*Pecten maximus*) and Frozen Atlantic Sea Scallops (*Placopecten magellanicus*) on a Retail Level

**DOI:** 10.3390/foods4040524

**Published:** 2015-09-29

**Authors:** Monika Manthey-Karl, Ines Lehmann, Ute Ostermeyer, Hartmut Rehbein, Ute Schröder

**Affiliations:** Department of Safety and Quality of Milk and Fish Products, Max Rubner-Institut, Federal Research Institute of Nutrition and Food, Palmaille 9, 22767 Hamburg, Germany; E-Mails: Ines.Lehmann@mri.bund.de (I.L.); Ute.Ostermeyer@mri.bund.de (U.O.); hum.rehbein@t-online.de (H.R.); Ute.Schroeder@mri.bund.de (U.S.)

**Keywords:** scallop products, chemical composition, additives, moisture to protein ratio, species identification, citric acid, phosphates, water content

## Abstract

An enlarged range of scallop products on the market allows the consumer to buy lower priced alternatives, which often raises the question of quality and control. Frozen meat of king scallops (*Pecten maximus*) and Atlantic sea scallops (*Placopecten magellanicus*) were purchased on the German market and compared with fresh shell-on king scallops of various origin. The approximate composition, inclusive citric acid and phosphates, minerals, free amino acids (FAA) and fatty acid profiles were examined in the muscle to identify changes as a result of processing. The FAA glycine and taurine as well the fatty acids 20:5n-3 (EPA) and 22:6n-3 (DHA) were the most abundant, but were reduced in processed samples. Di- and triphosphate contents were not detectable (<0.01 g·kg^−1^) in untreated meats. Most frozen scallop products contained added citrates and polyphosphates and had distinctly higher water contents (up to 89%) and an increased moisture to protein ratio (M/P) (up to 9) compared with the fresh king scallops (78%, M/P < 5). Labelling of species, verified by PCR-based DNA analysis, and ingredients were not correct in each case. Overall results indicated no relevant differences in mineral content, except high sodium contents, resulting from additives. Labelling does not readily allow the consumer to recognize the extent of processing effects.

## 1. Introduction

The initial quality of the raw material and changes during transport, processing and storage are key criteria for the assessment of seafood products. However, shifts in the biochemical composition, and also manipulations are mostly invisible to the consumer. The knowledge of those parameters which determine the original composition and the most important attributes can be used to identify adulterations, resulting in a loss of food quality.

Seafood, including shellfish, is considered as a healthy product in human diet. The main beneficial aspects derive from the nutritional value of the essential amino acids, highly digestible proteins, minerals and a high content of long chain polyunsaturated fatty acids.

The European market share of marine bivalve mollusks of the family Pectinidae, commonly known as scallops, has increased significantly in recent years and has been extended besides the king scallop (*Pecten maximus*) to a variety of other species, mainly the Atlantic sea scallop (*Placopecten magellanicus*). Scallops are marketed either in shells or as shucked adductor muscle meats after removing shells and intestine. They are sold fresh or deep frozen.

Wild-caught Atlantic sea scallops are grown in the Northwest Atlantic Ocean, from Newfoundland to North Carolina. They are mostly shucked on board and their meat is kept chilled, until delivered to shore side processors [1]. In 2012, the total catch was 267,745 ton [2].

In Europe, two members of the scallop genus *Pecten* are sold as king scallop or great Atlantic scallop: *Pecten maximus* is largely an Atlantic species, whilst *Pecten jacobaeus* is almost completely confined to the Mediterranean waters, despite a slight overlap in the Western area [3]. They are caught by dredging and other techniques, but also collected by divers as in Norway. Normally, further processing is done on shore. In total, about 64,000 t have been harvested in 2012 [2].

Scallops gained great prestige. However, frozen products are often associated with excessively high water contents [4,5,6]. The analysis of the so-called “added water” is a complex issue. The proximate composition of scallops shows some seasonal variations which are associated with the reproductive cycle. In general, the variations in moisture and protein are relatively low, but high in glycogen, fat and ash [7]. Between April and September the moisture content of Atlantic sea scallops range between 75% and 80%. Depending on the manner how they are treated during storage on board or during further processing steps, additional water may enhance the natural moisture content [8].

The addition of polyphosphates improves the water retention during processing [8,9] and may lead to an unjustified water uptake and increase in weight. Various phosphates have been widely accepted as additives in frozen fish and seafood. However, consumer’s growing interest in compositional aspects and the reservation against ingredients on chemical basis like phosphates lead to an increasing demand for “chem-free” clean labels. In response, additives like citric acid and its salts were introduced, sometimes in combination with bicarbonate salts which are difficult to detect. The new EU Food Information Regulation 1169/2011 [10], which entered into force in December 2014, provides a more transparent labelling and improves the consumer protection against hidden water. In addition, the Codex Committee on Fish and Fishery Products has addressed this matter and a Codex Standard for raw, fresh and quick frozen scallop products will be adopted the near future (REP 14FFP) [11]. Water addition with or without phosphates will only be accepted in raw quick frozen scallop meat products, if the “added water” is accurately measured and labelled as a part of the product name. Consequently, the draft Codex standard demands scientifically based criteria for the natural moisture level in the meat of harvested scallop species. The moisture (water) to protein ratio appears as suitable instrument to detect such excessive water, being more precise and reliable than the water content itself [6,12].

In order to verify the conformity with legislation and to comply with correct labelling, it will be a prerequisite for manufacturers and inspection authorities to know the natural composition of scallop muscles.

The aim of this study was to make a comprehensive overview of the composition of different scallop meat products on the German market and to evaluate possible indicators for added water. The muscle meat of whole fresh king scallops and various frozen products from retail were analyzed for the most important quality parameters, such as proximate composition, minerals, fatty acids and free amino acids.

## 2. Experimental Section

### 2.1. Sample Procurement

Fresh scallops, harvested from French fishing vessels off the Brittany (designated as France I) and Normandy coast (France II, III) in November 2011, as well as animals wild caught by a Norwegian company in FAO 27 (Norway I) in October 2011 or hand dived in the same period in Norwegian waters outside the islands of Frøya (Norway II) and Hitra (Norway III), were bought from different German wholesalers and delivered 4–7 days after capture in ice-cooled polystyrene containers to the Max Rubner-Institute in Hamburg, Germany. At the institute, they were manually shucked, and the adductor muscle, viscera and gonads, if any, were separated. To get enough material for all chemical analyses, 20 to 30 individual muscles were pooled and homogenized. Additionally, 10 individual specimens from France (I) and Norway (III) were prepared analogously, and analyzed one by one for proximate composition. All homogenates were kept deep frozen at −30 °C until analyzed.

Frozen scallop meat products were purchased in supermarkets and retail stores in 2011 and 2012. The adductor muscles were deglazed, following the instructions of the Codex method for quick frozen shrimps or prawns [13]. In brief, a deep frozen meat sample was weighed; the ice layer was stripped off by hand from the sample immersed in water, which is completed by feeling the slightly rough texture of the surface. The still frozen product was removed from the water bath and dried by use of a paper towel, before estimating the net product weight by a second measurement (weight difference = glaze content).

Pooled deglazed samples of 400–500 g (20–30 scallops) were thawed in a plastic sieve overnight at 4 °C. The drip loss was weighed and recombined with the samples before homogenization.

### 2.2. Chemical Analyses

All assays were conducted in replicate samples of the homogenates.

#### 2.2.1. Proximate Composition and pH

Percent moisture and ash content were determined by drying samples of approximately 5 g to constant weight at 105 °C for 12 h, followed by ashing at 550 °C [14].

The nitrogen content was obtained by a modified Dumas method, using a LECO model TruSpec N nitrogen analyzer (LECO Instruments GmbH, Mönchengladbach, Germany). Percent protein was calculated by multiplying percent nitrogen by 6.25 [15]. Total lipids were measured using the method described by Smedes [16] with modification by Karl *et al.* [17], which included extraction of lipids from the homogenized sample with a mixture of isopropanol and cyclohexane. After addition of water, the lipid-containing organic phase was separated, evaporated, dried and weighed. Percent salt (NaCl) was obtained by potentiometric titration of an aqueous sample solution with 0.1 N AgNO_3_ solution, applying the method of Karl *et al.* [18]. The total phosphorus content was estimated photometrically in the nitric acid extract of the ash, according to a modified official German method § 64 LFGB to determine phosphorus in meat [19]. Total carbohydrate concentration was analyzed by the phenol sulfuric acid method, according to Dubois *et al.* [20]. The colorimetric reaction was measured at 490 nm.

The pH value was determined in the minced samples with one part of deionized water.

#### 2.2.2. Citric Acid

For the citric acid determination an own laboratory procedure was developed. Following application recommendations of the company Phenomenex (supplier for chromatographic products, Aschaffenburg, Germany) for organic acids, we performed isocratic HPLC on the column Synergi Hydro RP 80A, 4 µm (250 × 4.6 mm) with the pre-column AQ C18 (4 × 3.0 mm) (both Phenomenex), and UV detection at 220 nm. The mobile phase consisted of 20 mM potassium dihydrogen phosphate, pH = 2.5, the flow rate was 0.4 mL·min^−1^. From the homogenized scallop samples (5 g) aqueous extracts (100 mL) were prepared by the addition of Carrez I and II (2 mL, respectively) to precipitate proteins. After filtration with a pleated filter, the extracts were further purified by syringe filters (0.2 µm) and analyzed (20 µL). The citric acid concentration of a sample was calculated by an extern calibration curve of citric acid standards with dilutions of 2.5 µg/mL to 200 µg/mL. Recovery rates for different fishery products are in the range of 80% to 104%. The limit of determination (LOD) for citric acid is 5 mg·kg^−1^.

#### 2.2.3. Condensed Phosphates

Condensed phosphates as di- and triphosphates were analyzed by ion chromatography and conductivity detection by means of a suppressor technique, largely following the method of Kaufmann and coworkers [21,22]. The existing HPLC system did not allow using potassium or sodium hydroxide solutions without causing damage. Therefore, the method was based on a gradient elution with 50 mM sodium carbonate (*w*/*v*) and 50 mM sodium hydrogen carbonate buffer. As stationary phase the analytical column Metrosep A Supp 5–100 (Metrohm, Filderstadt, Germany) combined with two pre-columns, Metrosep A 4/5 Guard (Metrohm) and Hypercarb (4.6 × 10 mm, 5 µm; Fisher Scientific, Schwerte, Germany), were used. The preparation in brief: in a 250 mL centrifuge tube 5 g homogenized sample were heated with 146 mL deionized water at 100 °C for a few minutes to stop the phosphatase activity, centrifuged and filtered (syringe filters 0.2 or 0.45 µm) before injection (10 µL) into the HPLC-system. The LOD for di- and triphosphates is 10 mg·kg^−1^.

#### 2.2.4. Total Volatile Basic Nitrogen (TVB-N)

Based on the EU-method [23], TVB-N was estimated in an aliquot of a filtered perchloric acid extract of 20 g homogenized scallop muscle and 180 mL 6% (*w*/*w*) perchloric acid, using an automatic distillation apparatus (Vapodest 50, Königswinter, Germany). After liberation with 20% (*w*/*v*) NaOH, followed by steam distillation, the volatile base components were absorbed in 0.1% (*w*/*v*) boric acid solution and determined by titration with 0.01 N HCl.

#### 2.2.5. Fatty Acid Profiles

Fatty acid composition was determined according to the DGF standard method [24] with gas chromatography: Fatty acid methyl esters (FAME) were obtained from the extracted lipids by trans-esterification with potassium hydroxide in methanol [25]. Analyses were performed on an Agilent 7890 gas chromatograph (Agilent Technologies equipped with split injection port, autosampler, FID) and a 60-m fused silica capillary column (i.d.: 0.32 mm) coated with 0.25 μm of DB-23 (Agilent J&W). Internal standard: Nonadecanoic acid C19:0. Fatty acids in the range of 14:0–22:6 n-3 were estimated and represented as a percentage of all measured fatty acids. [26].

#### 2.2.6. Free Amino Acids

Free amino acids (including taurine) were analyzed according to a modified HPLC method by Antoine and co-workers [27]. For deproteinisation 10 g scallop muscle were homogenized with 90 mL 6% perchloric acid (*w*/*w*) and subsequently filtrated. HPLC determination of the free amino acids was performed in the diluted extracts (1:10 up to 1:2000). After pre-column derivatization with o-phthaldialdehyde (OPA), the 18 amino acids were separated on the reversed-phase column Nucleodur 100-5 C18 ec (250 × 4 mm) with the corresponding pre-column (3 × 4 mm) (Macherey-Nagel, Düren, Germany) by a solvent gradient and then quantified by fluorescence detection, using the internal standard method with 2-aminobutyric acid [28]. The limit of quantification was 1 mg/100 g scallop tissue for each amino acid.

#### 2.2.7. Mineral Element Analysis

Details are described by Karl *et al.* [29]. In brief, 2 g muscle homogenate were digested in a mixture of 4 mL 65% nitric acid (*w*/*w*) and 1 mL 30% hydrogen peroxide (*w*/*w*) in a closed tetrafluormethaxil quartz vessel of a temperature time programmed Milestone ultraCLAVE III digestion system (Milestone SRL, Sorisole, Italy).

Sodium (Na), potassium (K), Calcium (Ca), magnesium (Mg) and zinc (Zn) were measured by flame AAS (contrAA^®^ 700 high-resolution continuum source atomic absorption spectrometer with air-acetylene flame, equipped with an autosampler; Analytik Jena, Jena, Germany). Selenium (Se) and total arsenic (As) were analyzed by the continuous flow hydride system of the same device. For the reduction of Se (VI) to Se (IV) prior to the hydride generation, 6 M HCl was added to an aliquot of the sample solution (1:1, *v*/*v*) and heated in a water bath for 30 min at 90 °C. In order to determine the total arsenic concentration, a mixture (1:1, *v*/*v*) of 5% KJ/5% l(+)-ascorbic acid (*w*/*v*) has been employed as pre-reducing agent.

A commercial reference material (IAEA-407, International Atomic Energy Agency, Vienna, Austria) was used to validate the analytical methods and as quality control. The mean values obtained for analytical recovery were 81% (Na), 93% (K), 89% (Ca), 87% (Mg), 88% (Se), 87% (As) and 89% (Zn), respectively.

### 2.3. DNA Analysis

Extraction and quantification of DNA were performed as described previously [30] with the exception of the incubation time, which was extended from 1 h to overnight.

DNA analysis was based on two PCR methods: (i) First the scallops were screened by PCR-RFLP (restriction fragment length polymorphism) to examine, if samples of the same label gave the same DNA fragment pattern. A sequence of nuclear ribosomal DNA containing two internal transcribed spacers (ITS) was amplified and digested by *Alu I* according to López-Piñón *et al.*, 2002 [31]. (ii) The second step was to identify the different scallops by amplification and sequencing a section of the mitochondrial 16S rRNA gene [31].

PCR reactions were carried out with Hotstart Taq *Plus* Master Mix Kit (QIAGEN, Hilden, Germany) according to manufacturer’s instructions. In both PCR assays the concentration of the DNA was adjusted to 1 ng·µL^−1^ and the concentration of the primers to 0.5 pM·µL^−1^. PCR conditions were: preheating 15 min/95 °C, followed by 35 cycles of 1 min/94 °C, 1 min/55 °C, 1 min/72 °C, and final heating for 7 min/72 °C. Results of PCRs were checked by agarose gel electrophoresis with 2% agarose and staining with ethidium bromide, diluted to a final concentration of 0.1 µg·mL^−1^.

*RFLP*- *analysis of ITS*: Without purification the amplicons were digested by *Alu I* (Thermo Scientific, Schwerte, Germany) at 37 °C overnight; then the enzyme was inactivated by heating for 5 min at 85 °C. DNA fragments were separated by polyacrylamide gel electrophoresis using a CleanGel 15% (EDC, Tübingen, Germany) as described recently [32].

*Sequencing of 16S rRNA*: Amplicons were cycle-sequenced in both directions with the same primers as taken for PCR using the ABI Prism Big Dye Terminator Cycle Sequencing Ready Reaction Kit (Applied Biosystems^®^ Version 1.1, Thermo Scientific). DNA sequences were compared with nucleotide sequences from GenBank [33], by means of the programme BLAST (Basic Local Alignment Search Tool).

### 2.4. Statistical Evaluation

One-way ANOVA tests were applied for statistical analysis (SigmaStat version 3.5, Systat software Inc., San Jose, CA, USA).

## 3. Results and Discussion

### 3.1. Authentication

Before characterizing the composition of different scallops, the identification of species was clarified to exclude an erroneous labelling. The analyzed samples originate from aquaculture as well as from wild harvest and were named as *Pecten maximus*, *Placopecten magellanicus,* *Mizuhopecten yessoensis* or just *Pecten* spp. With the exception of fresh bivalves, morphological characteristics as shells are missed in the frozen meat products. Even traders, sellers and consumers with deep seafood knowledge will not be able to distinguish between the normally more expensive *P. maximus* or other scallop species. Consequently, DNA methods have to be applied for authentication.

**Figure 1:**
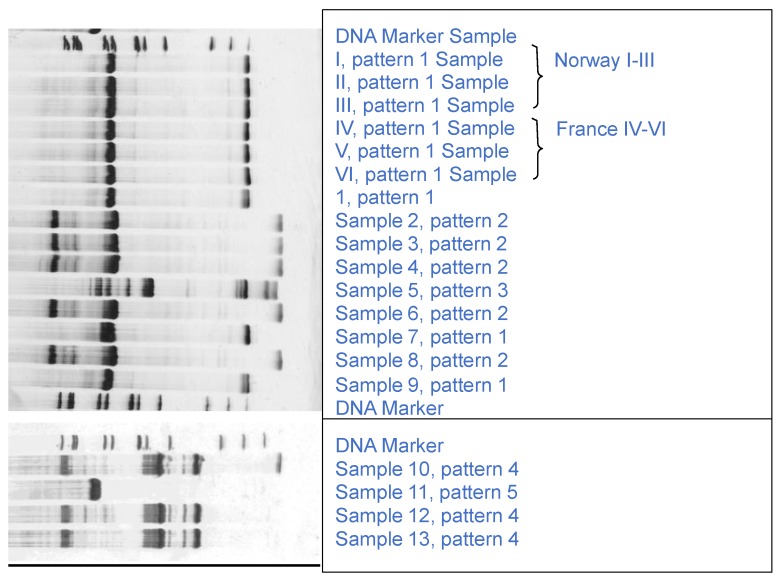
Fresh and frozen scallop samples: RFLP analysis of the ITS amplicons, digested by *Alu* I. Clean Gel 15%, silver staining. Samples I-VI: fresh king scallops (*Pecten maximus*), samples 1–13: frozen scallop meat products identified as king scallops (*P. maximus/ P. jacobaeus*) (1, 7, 9), Atlantic sea scallops (*Placopecten magellanicus*) (2, 3, 4, 6, 8, 10, 12, 13) and Japanese scallop (*Mizuhopecten yessoensis*) (5), used Marker was 100 bp DNA ladder (Roth, Germany).

The RFLP analysis of the ITS 2 fragment gave 5 different DNA fragment patterns (Figure 1). The samples I to VI, 1, 7 and 9 possessed the same pattern (pattern 1) with fragment lengths of 648 bp and 118 bp and could be assigned to the species *Pecten maximus* [34], confirming the declaration of these samples. Pattern 2 was found in case of samples 2–4, 6 and 8, which were labelled as *Placopecten magellanicus* respectively, except sample 2 (labelled as *Pecten* spp). Sample 5 and 11 gave unique patterns, whereas samples 10, 12 and 13 showed the same pattern (pattern 4). Based on the results of the screening by RFLP, following preliminary conclusion can be drawn: (i) Declaration of sample I-VI, 1, 7 and 9 was correct (*P.* *maximus*); (ii) Samples 2 and 13 have been mislabelled, because their pattern was different to *P.* *maximus*; (iii) Samples 2–4, 7, 8, 10 and 12, declared as *P. magellanicus*, expressed two different patterns (pattern 2 and 4).

The results of sequencing and BLAST of the 16S rRNA amplicon assigned samples III to VI, 1, 7 and 9 to *P. maximus* or *P. jacobaeus*, which cannot be distinguished by this method [35]. According to Canapa *et al.* [36], it has not been clarified until now, if they are two species or just different populations. The species *P. magellanicus* was detected in samples 2–4, 6, 8, 12 and 13, corroborating that sample 2 and 13 had not been correctly labelled. Sample 5 was identified as *Mizuhopecten yessoensis,* whereas no readable sequences were obtained for sample I, II, 10 and 11.

### 3.2. Chemical Results for Fresh King Scallop Meats (Pecten maximus)

#### 3.2.1. Proximate Analyses, including Moisture to Protein Ratio, pH, TVB-N and Mineral Content

Results are summarized in Table 1. The muscle pH value of all groups ranged between 6.0 and 6.2. It is known that the initial neutral pH can decrease as result of transportation stress. Comparable values were found in the fresh adductor muscle of the lion’s paw scallop *N. subnodosus* transported and stored whole in refrigeration conditions (3–6 °C) by Jiménez-Ruiz *et al.* [37]. In the same study TVB-N-values between 7.0 and 13.9 mg/100 g were obtained up to day 4 of refrigerated storage.

**Table 1:** Proximate composition of fresh king scallops (wet weight) from different harvest areas, Norway (I-III) and France (I-III): mean values for pooled samples of n individuals and mean values ± standard deviation for *n* = 10 individually analyzed specimens (results in brackets).

	Norway I (*n* = 20)	Norway II (*n* = 18)	Norway III (*n* = 20 + 10)	France I (*n* = 30 + 10)	France II (*n* = 20)	France III (*n* = 20)
Muscle weight (g)	27.0	38.3	24.7	17.3	26.1	23.7
pH	6.1	6.0	6.2	6.2	6.0	6.2
TVB-N (mg/100 g)	14.3	11.1	12.6	6.5	(24.5) ^1^	9.9
Moisture (%)	78.2	78.0	77.3 (76.2 ± 0.9)	76.5 (75.0 ± 1.1)	74.9	74.6
Protein (%)	18.9	19.2	18.2 (18.6 ± 0.5)	20.0 (18.9 ±0.8)	18.0	18.4
Moisture/Protein-Ratio	4.1	4.1	4.3	3.8	4.2	4.1
Lipid (%)	1.1	1.3	1.1	1.0	0.4	1.1
Carbohydrate (%)	2.7	3.2	4.0 (3.9 ± 0.7)	3.5 (3.5 ± 0.7)	6.8	6.7
Ash (%)	1.4	1.4	1.4 (1.8 ± 0.1)	1.5 (1.6 ± 0.2)	1.5	1.4
NaCl (%)	0.48	0.34	0.44	0.37	0.47	0.34
P_2_O_5_ (g kg^−1^)	6.3	5.8	5.8	6.0	6.0	6.3
Na (mg kg^−1^)	1028	1096	994	916	1167	826
K (mg kg^−1^)	3976	4047	3937	4118	3860	4100
Ca (mg kg^−1^)	301	278	296	267	255	240
Mg (mg kg^−1^)	342	352	330	356	348	350
Zn (mg kg^−1^)	13.7	13.3	15.1	16.9	12.6	13.3
Se (mg kg^−1^)	0.32	0.33	0.26	0.29	0.22	0.20
As (mg kg^−1^)	2.23	1.54	2.05	1.78	1.52	1.51
Citric acid (mg kg^−1^)	<5	<5	<5	<5	<5	<5
*Most frequently free amino acids (FAA)*						
Alanine (mg/100 g)	64	52	63	90	110	150
Arginine (mg/100 g)	180	265	280	365	240	325
Glycine (mg/100 g)	1520	1160	1640	1220	1580	1590
Taurine (mg/100 g)	795	890	975	1220	800	840
Total FAA (mg/100 g)	2666	2450	3053	3032	2840	2999

^1^ Samples were improperly cooled during transport and spoiled.

The analytical results for the adductor muscle of fresh king scallops are presented in Table 1 and were within the ranges reported for this species [38]. During catch/harvest season the variation between specimens on offer from different harvesting areas seems to be small.

The differences in lipid, protein and carbohydrate contents between the sample groups were not statistically significant (all *p* = 0.416). As expected, the lipid content was generally low and ≤1.3%. The mean moisture and protein values varied from 74.6% to 78.2% and from 18.0% to 20.0%, respectively. Analyses of individual scallops (Norway III, France I) also confirmed a reasonable degree of uniformity with low standard deviation. Calculated from all these data, the moisture to protein ratios were similar and all <5.0 (*p* = 0.416).

As consequence of different postharvest practices and a frequently inadequate labelling, the acceptable upper limit of natural moisture content has been discussed for years. Recommendations for the composition of processed scallops derived from moisture contents <80% [39,40] and a moisture/protein (M/P) ratio ≤5.0 [3,5]. All fresh samples fulfilled these guidelines.

There were no significant differences of the ash contents compared to other species [41]. Kimura [42] reported amounts between 1.5% and 1.6% for the Japanese scallop, *Mizuhopecten yessoensis*, whereby potassium (K) was the main ash’s macro element. Published data range from 4200 to 4800 mg·K·kg^−1^, which is slightly higher than the values shown in Table 1. Results for Na, Ca, and Mg were comparable. As expected for clams without additives, only small differences were found in the NaCl contents determined by titration with silver nitrate (mean value: 0.4%) and those amounts calculated from the Na content estimated by AAS (mean value: 0.3%). The mean Mg content (346 ± 9 mg·kg^−1^) in scallop meat was higher than the mean Ca content (273 ± 24 mg·kg^−1^). Similar levels are also found in many types of marine fish [43].

The total phosphorus (P) content (calculated as P_2_O_5_) corresponded well with earlier findings for freshly caught *Pecten maximus* (between 5.4 and 7.0 g P_2_O_5_·kg^−1^ muscle, (Manthey-Karl, not published). Data reported by Sidwell *et al.* [44] summarized mean P_2_O_5_ values of 6.2 ± 0.9 g·kg^−1^ for not specified scallops (Pectinidae spp.), covering a range of 4.8 to 7.8 g·kg^−1^.

Zinc (Zn) has many important biochemical functions, in particular as cofactor to more than 300 enzymes involved in RNA and DNA metabolism and stabilization of cell membranes [45]. In a review summarizing recent publications, 20.7 mg Zn·kg^−1^ (4.2–34.0 mg·kg^−1^) for clams, excluding oysters, were reported [46]. Greig *et al.* [47] analyzed even higher concentrations in female scallop gonads. Nevertheless, amounts of 12.6–16.9 mg Zn·kg^−1^ scallop muscle, found in this study, are equally worth mentioning.

Selenium (Se) as an essential micronutrient also plays a vital role in human health and protects against damage from free radicals and reactive oxygen species [48]. Data of 0.1 to 1.0 mg·kg^−1^ are reported for edible parts of seafood with average contents of 0.4 mg Se·kg^−1^ [46]. Se concentration between 0.20 and 0.33 mg·kg^−1^ found in the Norwegian and French king scallop muscles were in a comparable agreement to findings for other sampling locations [49].

Seafood has an important impact on human arsenic (As) intake [50]. As yet, no harmonized maximum levels are set by the EU. The International Agency for Research on Cancer (IARC) listed inorganic arsenic as human carcinogen. Its organic compounds, which are not metabolized in humans, are expected as not classifiable as to their carcinogenicity to humans [51]. The latter are the most common forms of total arsenic in seafood, although crustaceans and mollusks show in general slightly higher percentage shares of inorganic As compared to fish [52,53]. In this study, total arsenic contents between 1.51 and 2.23 mg·kg^−1^ (w.w.) were analyzed which are in a comparable order of magnitude of previously reported values for mollusks, inclusive scallops, which cover a wide range from 0.2 mg·kg^−1^ [54] up to 5.0 mg·kg^−1^ [49].

#### 3.2.2. Citric Acid

The determination of naturally occurring citric acid concentrations in adductor muscles of fresh king scallops (*Pecten maximus*) shall be the base to assess whether scallops meat products are treated with the food additive citric acid/citrate or not (Table 1). In general, citrate is produced in the citric acid cycle. It seems reasonable that small quantities of citric acid are detectable in scallop meats. Only few publications are dealing with the natural physiological concentration of citric acid in fish and fishery products. In the lateral muscle of mudskipper (*Boleophthalmus boddaerti*) approximately 44 mg citric acid kg^−1^ were measured [55], and in the seminal milt plasma of various fresh water fish like *Perca fluviatilis*, *Salmo gairdneri* and *Coregonus lavaretus*. Piironen and Hyvärinen [56] found average concentrations from 5.49 to 45.13 mg citric acid/100 mL plasma. In the present study no relevant citric acid concentrations (HPLC method LOD <5 mg·kg^−1^) could be detected in adductor meat samples of *Pecten maximus*. This supported results for various fishery products of the Swiss State Laboratory of the Canton Bern [57,58]. They concluded that citric acid concentrations in processed frozen fish and crustaceans <0.1 mg·kg^−1^ must be of natural origin. However, it could not finally clarified, whether traces of citric acid were natural or from unintended transfer effects of citric acid residues during the production of these products. Taking into account some natural variations, it could be assumed that different fishery species and origin (muscle meat, milt, roe, *etc.*) will show different organic acid contents at low level.

#### 3.2.3. Fatty Acid Profile

The fatty acid (FA) composition is influenced by the dietary fatty acids and the species [59]. In Table 2 the results for the fatty acid compositions for fresh king scallops are presented. The polyunsaturated fatty acids (PUFA) made up around 55% of total analyzed fatty acids, dominated by eicosapentaenoic acid (EPA 20:5n3) and docosahexaenoic acid (DHA 22:6n3). Saturated fatty acids represented around 30%, mainly palmitic acid (16:0) and stearic acid (18:0).

**Table 2:** Average composition of fatty acids (FA) of fresh king scallop meat from different harvest areas (Norway I-III and France I-III) and of frozen king scallop and Atlantic sea scallop meat products (% of fatty acids measured; SFA = saturated fatty acids; MUFA = monounsaturated fatty acids; PUFA = polyunsaturated fatty acids; n.d. = not detected).

		King Scallops			Atlantic Sea Scallops
		Fresh		Frozen	Frozen
FA common name	FA Shorthand	Norway I-III *n* = 3	France I-III *n* = 3	*n* = 4	*n* = 8
Myristic acid	14:0	2.4 ± 0.02 ^a^	2.5 ± 0.20 ^a^	3.2 ± 0.36 ^b^	2.1 ± 0.20 ^a^
Pentadecanoic acid	15:0	0.8 ± 0.01 ^a^	0.8 ± 0.03 ^a^	0.8 ± 0.06 ^a^	0.8 ± 0.09 ^a^
Palmitic acid	16:0	17.3 ± 0.20 ^a^	18.4 ± 0.29 ^b^	17.4 ± 0.88 ^a^	18.6 ± 0.55 ^b^
Heptadecanoic acid	17:0	1.0 ± 0.03 ^a^	1.1 ± 0.04 ^a^	1.1 ± 0.11 ^a^	0.7 ± 0.09 ^b^
Stearic acid	18:0	7.1 ± 0.18 ^a^	7.9 ± 0.25 ^b^	6.8 ± 0.35 ^a^	5.6 ± 0.31 ^c^
	**∑SFA**	28.5 ± 0.30 ^a^	30.7 ± 0.47 ^a^	29.3 ± 1.12 ^a^	27.8 ± 1.20 ^a^
Palmitoleic acid	16:1n-7	1.6 ± 0.15 ^a^	1.2 ± 0.04 ^a^	1.4 ± 0.39 ^a^	2.0 ± 0.52 ^a^
Elaidic acid	18:1n-9t	n.d.	n.d	n.d	n.d
Oleic acid	18:1n–9*c*	1.0 ± 0.13 ^a^	1.0 ± 0.05 ^a^	1.3 ± 0.12 ^b^	1.2 ± 0.12 ^ab^
Vaccenic acid	18-1n-7	3.1 ± 0.32 ^a^	3.2 ± 0.30 ^a^	2.9 ± 0.25 ^a^	5.4 ± 0.24 ^b^
Gondoic acid	20:1n–9	1.7 ± 0.13 ^a^	1.5 ± 0.20 ^a^	1.8 ± 0.33 ^a^	0.9 ± 0.06 ^b^
Erucic acid	22:1n-9	n.d.	n.d.	n.d.	n.d.
	**∑MUFA**	7.3 ± 0.26 ^a^	6.8 ± 0.43 ^a^	7.4 ± 0.42 ^a^	9.4 ± 0.78 ^b^
Linoleic acid	18:2n–6*c*	0.4 ± 0.27 ^a^	0.4 ± 0.28 ^a^	0.5 ± 0.27 ^a^	0.4 ± 0.04 ^a^
γ-Linolenic acid	18:3n–6	n.d	n.d	n.d	n.d
α-Linolenic acid	18:3n–3	0.2 ± 0.21 ^a^	0.2 ± 0.27 ^a^	0.7 ± 0.04 ^b^	0.2 ± 0.18 ^a^
Stearidonic acid	18:4n–3	1.4 ± 0.13 ^a^	1.8 ± 0.12 ^ab^	2.1 ± 0.30 ^b^	1.7 ± 0.39 ^ab^
Eicosadienic acid	20:2n–6	0.7 ± 0.03 ^a^	0.7 ± 0.05 ^a^	0.3 ± 0.34 ^b^	0.3 ± 0.11 ^b^
Arachidonic acid	20:4n–6	3.4 ± 0.24 ^a^	3.4 ± 0.58 ^a^	2.2 ± 0.26 ^b^	1.8 ± 0.15 ^b^
Eicosapentaenoic acid (EPA)	20:5n–3	20.0 ± 0.79 ^ac^	18.0 ± 0.86 ^ac^	14.8 ± 0.95 ^b^	20.3 ± 0.98 ^c^
Docosatetraneoic acid	22:4n–6	0.1 ± 0.17	n.d	n.d	n.d
Docosapentaenoic acid (DPA)	22:5n–3	1.6 ± 0.14 ^a^	1.2 ± 0.23 ^ab^	1.0 ± 0.02 ^bc^	0.9 ± 0.06 ^c^
Docosahexaenoic acid (DHA)	22:6n–3	26.2 ± 0.68 ^a^	25.9 ± 0.46 ^a^	26.1 ± 1.84 ^a^	21.3 ± 1.78 ^b^
	**∑PUFA**	53.9 ± 0.45 ^a^	51.6 ± 1.20 ^a^	47.8 ± 2.56 ^b^	47.0 ± 1.08 ^b^
	unidentified	10.4 ± 0.60 ^a^	10.9 ± 0.30 ^a^	15.5 ± 2.16 ^b^	15.8 ± 0.63 ^b^
	∑n–3	49.3 ± 0.34	47.2 ± 0.85	44.7 ± 2.18	44.5 ± 1.05
	∑n–6	4.6 ± 0.17	4.4 ± 0.35	3.0 ± 0.59	2.5 ± 0.09
	Ratio n-3/n-6	10.9 ± 0.38	10.7 ± 0.62	15.2 ± 2.52	18.2 ± 0.44
	EPA + DHA	46.2 ± 0.23	44.0 ± 0.72	40.9 ± 2.23	41.6 ± 1.05

Different letters in the same row indicate a statistically significant difference in the mean values (*p* < 0.05).

There were no significant differences between the fatty acid composition of the grouped scallop samples which were in the same range as described earlier by Grahl-Nielsen [60]. Even between the two origins France and Norway only slight differences of some fatty acid contents were noticed. The contents of palmitic, stearic, and stearidic acid were marginally higher in the mollusks from French waters, palmitoleic, eicosapentaenoic and docosapentaenoic acids were insignificantly lower than in the scallops from Norwegian waters. Summarizing these findings, the values were approximately equal to each other. A distinction based on these parameters was not possible. Due to the low fat content, the concentrations per 100 g edible portion are not mentionable.

#### 3.2.4. Free Amino Acids

Free amino acids (FAA) are relevant to flavor, taste, and coloring of cooked foods. Seafood usually contains more FAA than terrestrial animals due to the osmoregulatory function of these compounds [61].

**Figure 2:**
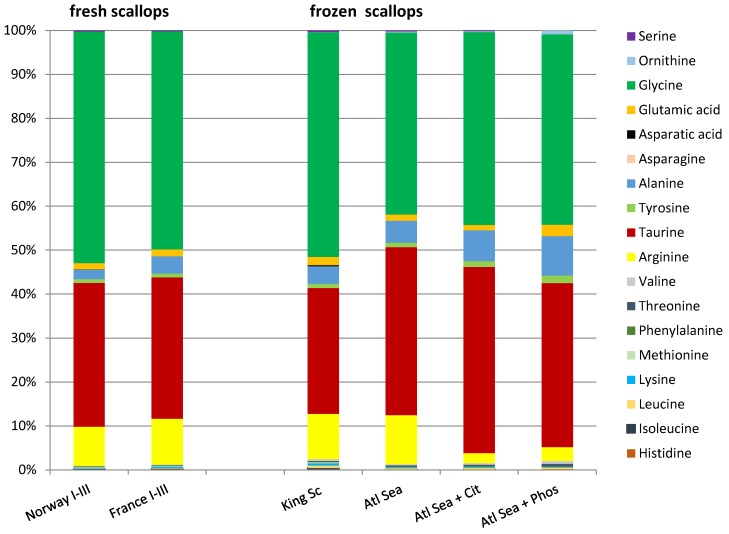
Average percentage composition of free amino acids in fresh king scallop meat (Norway I-III, France I-III), frozen king scallop meat (King Sc), and Atlantic sea scallop meat with either no additives (Atl Sea), or an addition of citrate (Atl Sea + Cit) or phosphate (Atl Sea + Phos).

The total FAA of all Norwegian and French samples ranged between 2.45 g and 3.05 g/100 g scallop adductor muscle (Table 1). Thus, these amounts are higher than those of many fish species (own results; [62]). The contents of the single analyzed FAA varied more or less among the pools of different origin. The most abundant FAA was glycine (40%–57%) (Figure 2). In addition, significant amounts of taurine (28%–40%), arginine (7%–12%), alanine (2%–5%), as well as glutamic acid and tyrosine (each with approximately 1%), were included. However, the levels of essential amino acids were low. The four acids glycine, arginine, alanine, and taurine accounted for 95.5%–96.9% of the total. Similar total contents and distributions of FAA were already found in other scallops like *Pecten albicans*, *Mizuhopecten yessoensis* or *Nodipecten subnodosus* [42,63,64,65]. Glycine and alanine contribute to the sweet taste of scallops. Both amino acids should have a synergistic effect in the mixture with glutamic acid; large amounts of arginine enrich their sweet taste [64]. On the one hand, arginine is of importance for the formation of various biologically important molecules [66]. On the other hand, arginine leads to the rapid formation of the biogenic amines agmatine and putrescine, produced by microorganism during cold storage of scallop products [67].

Muscle tissue of scallops contains a considerable amount of the amino sulfonic acid taurine that is much higher than in the edible part of most fish species [68]. Taurine is regarded as important for many physiological processes in humans. For instance, it is beneficial for cardiovascular health, cell membrane stabilization and immune defense enhancement; it reduces blood cholesterol values and has antioxidant properties [66,69]. Comparably high taurine concentrations of nearly 900 to 1100 mg/100 g adductor muscle of *Mizuhopecten yessoensis* are reported [42].

### 3.3. Products of Frozen King Scallops and Atlantic Sea Scallop Meats

#### 3.3.1. Proximate Analyses, including pH, TVB-N-Values and Mineral Elements

Table 3 shows the results divided in different treatment groups with/without additives. Mean moisture values for all frozen scallop products were higher compared to the analyzed fresh king scallops, whereas all protein values were lower. The use of additives led to an additional upward and downward shift, respectively. Elevated pH values were associated with such treatments. Lipid, carbohydrate, and ash contents were within the range expected. TVB-N-values were comparable to those of fresh scallops of good quality. All Atlantic sea scallop meats contained more Na, covering a large range. Except for Ca, the other minerals and trace elements were reduced compared to fresh scallops.

**Table 3:** Composition of frozen meat products of king scallops and Atlantic sea scallops with no labelled information on additives (NA), or an addition of citrate E331 or E330/331 or phosphate E451, respectively (arithmetic mean ± standard deviation; minimum-maximum amount) ^1^.

	King Scallops	Atlantic Sea Scallops		
Labelling (No of Products Analyzed)	NA (4)	NA (4)	Citrate (3)	Phosphate (1)
Glaze (%)/drip loss (%)	8-27/20-34	7-15/1-8	7-13/6-10	12/6
pH	6.4 ± 0.5 (6.0–7.0)	6.9 ± 0.6 (6.4–7.8)	8.0 ± 0.6 (7.6–8.7)	7.5
TVB-N (mg/100 g)	14.6 ± 1.5 (12.9–16.5)	17.8 ± 2.9 (13.7–20.4)	12.7 ± 3.2 (9.1–15.3)	13.4
Moisture (%)	81.2 ± 5.1 (76.4–86.7)	80.5 ± 4.3 (76.6–86.5)	86.8 ± 2.3 (84.5–89.1)	85.7
Protein (%)	14.1 ± 3.7 (10.2–16.6)	15.0 ± 3.6 (10.0–17.8)	10.5 ± 1.7 (9.2–12.4)	7.8
Moisture/Protein-Ratio	6.2 ± 2.0 (4.4–8.6)	5.7 ± 2.0 (4.3–8.6)	8.3±1.3 (6.8–9.2)	11.0
Lipid (%)	0.8 ± 0.1 (0.7–0.9)	0.6 ± 0.1 (0.4–0.7)	0.4 ± 0.1 (0.3–0.5)	0.5
Carbohydrate (%)	3.5 ± 1.2 (2.4–4.8)	2.9 ± 2.3 (0.4–5.8)	1.9 ± 0.4 (1.4–2.3)	4.6
Citric acid (g·kg^−1^)	<0.005	<0.005 -0.65	0.53–2.12	<0.005
Ash (%)	1.35 ± 0.58 (0.65–1.42)	1.69 ± 0.27 (1.42–2.05)	1.82 ± 1.17 (0.70–3.03)	1.93
NaCl (%)	0.29 ± 0.13 (0.16–0.42)	0.46 ± 0.14 (0.31–0.63)	0.25 ± 0.22 (0.11–0.50)	0.21
P_2_O_5_ (g·kg^−1^)	6.0 ±.2.3 (3.5–9.1)	6.9 ± 1.6 (5.9–9.3)	4.78 ± 5.43 (1.03–11.00)	9.7
Diphosphate (g·kg^−1^)	<0.01 (3) ^2^ –2.39	<0.01	<0.01	3.61
Triphosphate (g·kg^−1^)	<0.01 (3) ^2^ –0.93	<0.01	<0.01	1.88
Na (mg·kg^−1^)	1458 (240–3530)	2797 (1513–5636)	3760 (1658–5616)	4907
K (mg·kg^−1^)	2298 (1181–3515)	2902 (913–4135)	1099 (602–2004)	492
Ca (mg·kg^−1^)	450 (219–635)	388 (311–483)	486 (438–512)	486
Mg (mg·kg^−1^	268 (162–390)	322 (138–430)	140(106–205)	105
Zn (mg·kg^−1^)	16.5 (12.3–21.6)	10.3 (6.9–13.7)	8.5 (7.5–9.6)	5.3
Se (mg·kg^−1^)	0.2	0.2 (0.1–0.2)	0.1	0.1
As (mg·kg^−1^)	1.0 (0.6–1.3)	1.2 (0.5–1.9)	1.0 (0.5–2.1)	0.5
*Most frequently free amino acids (FAA)*				
Alanine (mg/100 g)	53 (38–82)	75 (25–113)	63 (39–100)	52
Arginine (mg/100 g)	156 (64–350)	249 (11–565)	16 (6–33)	18
Glycine (mg/100 g)	693 (453–1140)	688 (168–905)	381 (288–540)	251
Taurine (mg/100 g)	395 (235–675)	681 (144–1220)	385 (225–620)	216
Total FAA (mg/100 g)	1374 (842–2356)	1761 (367–2854)	883 (631–1321)	579

^1^ Some results for proximate composition have been published by Manthey-Karl *et al.* (2012) [6]. ^2^ Number of products.

#### 3.3.2. NaCl, Phosphates and Citric Acid

In recent years, an increasing number of frozen seafood with high water contents, high pH-values and bad sensory quality were found on the European market [70,71]. The chemical investigations in terms of all important components showed that these products were either treated with polyphosphates or probably with bicarbonates often in combination with citric acid or its salts [72].

Citric acid and its sodium salt are mainly used as additives to extend the shelf life of food, in particular fishery products. Acting as chelators, acidulants, and synergists of primary antioxidants, they show antibacterial effects, can bind off-flavor compounds and prevent lipid oxidation [73,74,75,76]. Sometimes citric acid is used to decrease the pH-value. Similarly to phosphates, sodium citrate as an ionic compound dissolves myofibrillar proteins even better than NaCl [77,78]. It promotes swelling and increases the water holding capacity. However, sodium citrate alone is not as effective as polyphosphates.

According to published research studies, bicarbonates in combination with small quantities of citric acid are powerful tools to achieve higher product weight by water addition [79]. Actually, no validated chemical method exists, distinguishing the added bicarbonate from the naturally present amount. However, an indirect prove of added sodium bicarbonate can be achieved by the determination of elevated sodium contents which do not fit to the sum of sodium derived from the natural and labelled ingredient sources.

##### Frozen King Scallops

According to the labelling, all frozen king scallop meat products of this study should be free of additives, except salt. The NaCl contents were between 0.2% and 0.4% (Table 3), corresponding to 800–1600 mg Na·kg^−1^. However, considering the maximum Na content of 3530 mg·kg^−1^ in one product, another Na-containing compound than salt must have been added. Additionally, the corresponding 9.1 g P_2_O_5_·kg^−1^ and the pH of 7.0 were the highest values in the king scallops group. Regarding the average content of 6.0 g P_2_O_5_·kg^−1^ in the fresh king scallop adductor muscles (Table 1), the distinctly higher content of 9.1 g P_2_O_5_·kg^−1^ probably derived from the addition of condensed phosphates during processing. Ion chromatography confirmed the presence of diphosphate and triphosphate in concentrations of 2.4 g·kg^−1^ and 0.9 g·kg^−1^ (calculated as P_2_O_5_), respectively. The moisture (84.4%) to protein (11.9%) ratio was 7.1. In this product the treatment with a food additive was fraudulently concealed. However, due to the natural variability, the determination of the total phosphorus content alone is not appropriate to prove a hidden polyphosphate addition.

##### Frozen Atlantic Sea Scallops

Products of frozen Atlantic sea scallop meats (Table 3) were either labelled as free of additives or as processed with citric acid (E 330) and/or monosodium citrate (E331) as well as sodium triphosphate (E 451). In one case citric acid was not labelled, although the concentration of 0.65 g·kg^−1^ in combination with 86.5% moisture (and 10.0% protein) indicated a technological effect in the product. Another Atlantic sea scallop meat product contained high levels of polyphosphates (3.6 g diphosphate (P_2_O_5_) kg^−^^1^ and 1.9 g triphosphate (P_2_O_5_) kg^−1^) and exceeded the legal limit of 5.0 g P_2_O_5_·kg^−1^. The associated increase up to 11 of the moisture/protein-ratio revealed an excessive water addition (85.7% moisture, 7.8% protein) and consumer deception.

The variation of the salt (NaCl) content was high (0.11%–0.63%). Some producers labelled the use of salt on the ingredient list. Salt contributes to the increase of water holding capacity [80]. In the group of Atlantic sea scallops without additives, products with 0.5%–0.6% NaCl, had only a drip loss of 1%–2%, whereas meats with slightly lower 0.3% to 0.4% NaCl had drip losses of 6 to 8%. Products treated with citric acid and phosphate had a comparable drip loss after thawing, but it can be assumed that the water holding capacity will be maintained better during cooking [81].

#### 3.3.3. Fresh *versus* Frozen Scallops: Moisture to Protein Ratio

The range for the analyzed naturally occurring organic and inorganic minor elements in fresh or frozen meat was similar (*p* < 0.05). It seemed that different processing practices after harvest did not eliminate desired nutritional components. However, even considering the natural variation within a species due to biological conditions, the considerable difference between fresh and frozen scallops was the uptake of water and as consequence the reduction of proteins.

No international agreed definition to the question “what is added water” exists, but one is the French specification which requires a ratio of moisture to protein (M/P) content ≤5 [4] and is one of the most simple and best methods to detect extraneous moisture. In our study, the quotient of all samples with water content <80% was as well ≤5 (Table 1). Our findings agreed to the French opinion that the M/P ratio is an appropriate indicator of water addition in scallop meat, including a toleration of 5%.

#### 3.3.4. Fatty Acid Profile

Table 2 shows the average composition of fatty acids in the different scallop samples. Some fatty acids differed in their contents between the fresh king scallops and the frozen king and Atlantic sea scallop products.

Slightly higher amounts of 14:0, 18:1n-9c and 18:4n-3 were found in the frozen king scallops compared to the fresh ones. The low amount of fatty acid 20:5n-3 (EPA) seems to correspondent with the percentage of unidentified FA, but a scientific justification cannot be given in this study. The FA composition of frozen Atlantic scallop meats was comparable to fresh king scallops with the exception of DHA, which was considerably lower.

#### 3.3.5. Free Amino Acids

The total amounts of free amino acids in frozen king scallops were in part significantly below the levels that were determined in the fresh specimens (Table 3) and have obviously been decreased by leaching and uptake of water during intensive immersion in water. However, the percentage compositions of FAA were similar for fresh and frozen king scallops (Figure 2).

The contents of single FAA varied considerably between samples (Table 3). As with the king scallops, even in all Atlantic sea scallops glycine, taurine, arginine and alanine were quantitatively the most highly represented FAA (Figure 2).

Due to the increased moisture content, Atlantic sea scallop meat with added phosphates or citrates contained on the average considerably lower percentage FAA than such products without additives.

## 4. Conclusions

Scallops and the products thereof are seen as a tasty low fat and protein rich food product by the consumer. Despite a natural variability, chemical parameters measured showed a balanced composition which also contains desired compounds like taurine, EPA and DHA as well as zinc or selenium. However, with increasing moisture content their percentage share decreased.

Consumer fraud by water addition to seafood products is an increasing problem in European seafood trade. Our actual survey on the German market revealed that many frozen scallop products contained more water than is acceptable, which offers an economic advantage by enhancing the product yield. As water binding additives, a combination of sodium bicarbonate/citric acid and salt is used, which increasingly replace polyphosphates. The investigation shows that the protein to moisture ratio can be considered as a simple method for the detection of water addition, if values > 5 are found. Such products were associated with a pH > 7.0.

Scallops have typically about 75%–80% water and a pH of 6.0–6.2, when they are freshly harvested from the ocean. Some manufacturers appear to add more than 5% water, making customers pay for it instead of scallop meat. The increase of moisture does not look impressive. However, it is only a simple calculation: A moisture content > 85% in scallops is the result of 30% water addition which is a good deal, because nothing is cheaper than air or water.

Apart from the influence of the processing conditions on deep frozen scallop meat products, the labelling of the species was not correct in every case. In some products, king scallops, having a high regard by the German consumer, were substituted by other species. A clear and transparent labelling should allow the consumer to make an informed choice.
